# Glycerate kinase of the hyperthermophilic archaeon *Thermoproteus tenax*: new insights into the phylogenetic distribution and physiological role of members of the three different glycerate kinase classes

**DOI:** 10.1186/1471-2164-8-301

**Published:** 2007-08-31

**Authors:** Daniel Kehrer, Hatim Ahmed, Henner Brinkmann, Bettina Siebers

**Affiliations:** 1 Department of Biology and Geography, Institute of Biology, Microbiology I, Universität Duisburg-Essen, Universitätsstr. 5, 45117 Essen, Germany; 2 Département de biochimie, Faculté de Médecine, Université de Montréal, C.P. 6128, Succ. Centre-ville, Montréal, Qc H3C 3J7, Canada

## Abstract

**Background:**

The presence of the branched Entner-Doudoroff (ED) pathway in two hyperthermophilic Crenarchaea, the anaerobe *Thermoproteus tenax *and the aerobe *Sulfolobus solfataricus*, was suggested. However, so far no enzymatic information of the non-phosphorylative ED branch and especially its key enzyme – glycerate kinase – was available. In the *T. tenax *genome, a gene homolog with similarity to putative hydroxypyruvate reductase/glycerate dehydrogenase and glycerate kinase was identified.

**Results:**

The encoding gene was expressed in *E. coli *in a recombinant form, the gene product purified and the glycerate kinase activity was confirmed by enzymatic studies. The enzyme was active as a monomer and catalyzed the ATP-dependent phosphorylation of D-glycerate forming exclusively 2-phosphoglycerate. The enzyme was specific for glycerate and highest activity was observed with ATP as phosphoryl donor and Mg^2+ ^as divalent cation. ATP could be partially replaced by GTP, CTP, TTP and UTP. The enzyme showed high affinity for D-glycerate (K_m _0.02 ± 0.01 mM, V_max _of 5.05 ± 0.52 U/mg protein) as well as ATP (K_m _of 0.03 ± 0.01 mM, V_max _of 4.41 ± 0.04 U/mg protein), although at higher glycerate concentrations, substrate inhibition was observed. Furthermore, the enzyme was inhibited by its product ADP via competitive inhibition. Data bank searches revealed that archaeal glycerate kinases are members of the MOFRL (multi-organism fragment with rich leucine) family, and homologs are found in all three domains of life.

**Conclusion:**

A re-evaluation of available genome sequence information as well as biochemical and phylogenetic studies revealed the presence of the branched ED pathway as common route for sugar degradation in Archaea that utilize the ED pathway. Detailed analyses including phylogenetic studies demonstrate the presence of three distinct glycerate kinase classes in extant organisms that share no common origin. The affiliation of characterized glycerate kinases with the different enzyme classes as well as their physiological/cellular function reveals no association with particular pathways but a separate phylogenetic distribution. This work highlights the diversity and complexity of the central carbohydrate metabolism. The data also support a key function of the conversion of glycerate to 2- or 3-phosphoglycerate via glycerate kinase in funneling various substrates into the common EMP pathway for catabolic and anabolic purposes.

## Background

Modifications of the ED pathway, the semi-phosphorylative (sp) and the non-phosphorylative (np) ED pathway, have been identified in all three forms of life [1]. Initial biochemical studies in Archaea revealed the presence of the npED pathway in (hyper)thermophiles [2-8] and the spED pathway in halophiles [9]. However, a recent approach that combined comparative genomics and biochemistry identified the presence of the spED pathway – probably in addition to the npED pathway – in *T. tenax *and *S. solfataricus*, suggesting the presence of a branched ED pathway in these hyperthermophiles. [8,10-13]. The hyperthermophilic anaerobe *Thermoproteus tenax *grows optimally around 90°C, pH 5. This sulphur-dependent Creanarchaeon is able to grow chemolithoautotrophically on CO_2 _and H_2_, as well as chemoorganoheterotrophically in the presence of various organic compounds such as starch, glucose, malate and methanol [14,15]. Therefore, *T. tenax *represents a perfect model organism for studying the complexity of the central carbohydrate metabolism as well as its regulation.

**Figure 1:**
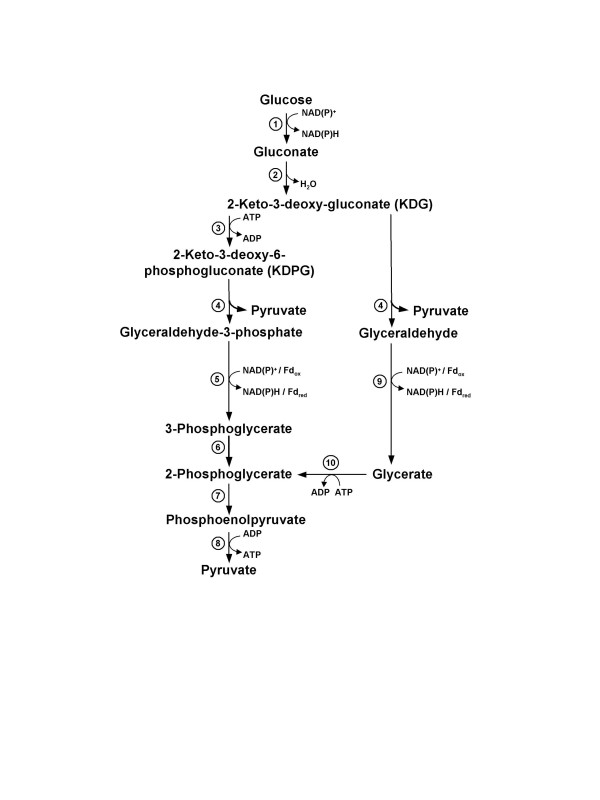
**Branched Entner-Doudoroff (ED) pathway in *Thermoproteus tenax***. The common ED shunt comprises the conversion of glucose into 2-keto-3-deoxygluconate (KDG), which is either phosphorylated and cleaved forming pyruvate and glyceraldehyde-3-phosphate (GAP) in the semi-phosphorylative ED branch (left side) or directly cleaved forming pyruvate and glyceraldehyde in the non-phosphorylative branch (right side). Enzyme key: 1, glucose dehydrogenase; 2, gluconate dehydratase (GAD); 3, 2-keto-3-deoxygluconate (KDG) kinase; 4, 2-keto-3-deoxy-(6-phospho)gluconate (KD(P)G) aldolase; 5, non-phosphorylating GAP dehydrogenase (GAPN) or GAP oxidoreductase; 6, phosphoglycerate mutase; 7, enolase; 8, pyruvate kinase; 9, aldehyde dehydrogenase or aldehyde oxidoreductase; 10, glycerate kinase.

In the suggested branched ED pathway [10], glucose is oxidized to gluconate via glucose dehydrogenase, and gluconate is dehydrated forming 2-keto-3-deoxygluconate (KDG) by gluconate dehydratase (GAD). In the spED branch, KDG is phosphorylated via KDG kinase, the key enzyme of the spED branch, and the formed 2-keto-3-deoxy-6-phosphogluconate (KDPG) is cleaved into glyceraldehyde-3-phosphate (GAP) and pyruvate by the action of the bifunctional KD(P)G aldolase, which is a key player in both ED branches. GAP is processed via the common lower shunt of the EMP (Embden-Meyerhof-Parnas) pathway, which is characterized by the presence of an unusual irreversible non-phosphorylating GAP dehydrogenase and/or GAP oxidoreductase in hyperthermophiles. These enzymes substitute for the anabolic enzyme couple NADP^+^-dependent GAP dehydrogenase and phosphoglycerate kinase [11,16-18]. In the npED branch, KD(P)G aldolase cleaves KDG into pyruvate and glyceraldehyde. Glyceraldehyde is further oxidized to form glycerate either by a NAD(P)^+^-dependent glyceraldehyde dehydrogenase [3,19,20] or by a ferredoxin-dependent glyceraldehyde oxidoreductase [5,21-23]. Glycerate is phosphorylated via glycerate kinase, the key enzyme of the npED branch, forming 2-phosphoglycerate. 2-Phosphoglycerate then enters the common lower shunt of the EMP pathway yielding a second molecule of pyruvate by the action of enolase and pyruvate kinase (Fig. 1).

Enzymes of the common ED shunt (glucose dehydrogenase [6,10,24,25], gluconate dehydratase [10,26,27] and KD(P)G aldolase [24,28,10]) as well as the spED branch (KDG kinase [10,29]) have been characterized from the hyperthermophilic Archaea *T. tenax *and *S. solfataricus*. Interestingly, these enzymes were shown to be promiscuous for glucose and galactose catabolism in *S. solfataricus *[24-26,28,29], which relies on the ED pathway as only route for sugar degradation. In contrast, the pathway seems to be specific for glucose and is active in addition to the EMP variant in *T. tenax *[6,10]. Glyceraldehyde oxidoreductase activity has been reported in cell extracts of *T. tenax *[5]. However, no information is currently available for the key enzyme of the npED branch, glycerate kinase, in *T. tenax *and hyperthermophilic Archaea in general. Studies addressing this metabolic key reaction are expected to reveal some new important insights into the regulation and the physiological role of the branched ED pathway.

Glycerate kinases have been characterized from all domains of life: Bacteria, Eukarya and Archaea. In general, two distinct classes of glycerate kinases were identified: 3-phosphoglycerate (3-PG) and 2-phoshoglycerate (2-PG) forming glycerate kinases. 3-PG forming enzymes were characterized for example in plants, fungi and different heterotrophic bacteria. 2-PG forming glycerate kinases, on the other hand, were identified and examined in animals and methylotrophic bacteria. Recently, the 2-PG forming glycerate kinases of the thermoacidophilic Euryarchaea *Picrophilus torridus *and *Thermoplasma acidophilum *were characterized and their function as key enzyme of the npED pathway in thermophiles was reported [30,32]. However, up to now little attention was given to the phylogenetic affiliation of the enzymes. Initial phylogenetic analyses published previously indicate the presence of three glycerate kinase families or groups and wrongly suggest that they share a common origin [31,32].

**Table 3:** Characterized and predicted members of the three different glycerate kinase classes

**Glycerate kinase class**	**Organism**	**Physiological function; Pathway**	**Reaction product**	**Literature**
**GK class II (MOFRL family)**	**Archaea**			
	*Thermoproteustenax*	Glucose degradation via sugar acids (gluconate); Branched ED pathway	2-PG	This manuscript, [30,32]
	*Picrophilustorridus*			
	*Thermoplsma acidophilum*			
	**Bacteria**			
	Facultative Methylotrophs, *Methylobacterium extorquens Hyphomicrobium methylovorum*	Growth on C1-compounds (e.g. methane, methanol) and conversion in C3-compounds via hydroxypyruvate; Assimilatory serine pathway	2-PG	[33,44-47]
	*Pseudomonas *sp.	Growth on C1 and C2-compounds (e.g. methanol, oxalate, glycolate); Serine pathway & glyoxylate metabolism^1^	-	
	*Agrobacterium vitis *(two plasmid-encoded genes, glycerate kinase instead of hydroxypyruvate reductase activity predicted)	Tartrate utilization, shares common reactions with the serine pathway; Tartrate utilization pathway	2-PG	[48,49]
	**Eukarya**			
	Rat liver, rat kidney cortex	Gluconeogenesis from serine, fructose metabolism	2-PG	[51-53]

**GK class I**	**Bacteria**			
	*Escherichia coli *(K12) GK-1	Allantoin assimilation (purine degradation); Glycerate pathway	3-PG	[55]
	*Escherichia coli *(K12) GK-2	Sugar acid degradation; Glucarate/galactarate utilization pathway	2-PG	[56]
	*Pseudomonas *sp.	Growth on sugar or sugar acids ^1^	-	
	*Flavobacterium *strains	Growth on ethylene glycol; Glycerate pathway	2-PG	[57]

**GK class III**	**Eukarya**			
	Plant, *Arabidopsis thaliana*	Photorespiration; C2-cycle	3-PG	[31]
	Fungi, *Neurospora crassa*	Growth on glycerol; Oxidative glycerol metabolism	3-PG	[58]

^1^the function of the class I and class II (MOFRL family) glycerate kinase for *P. fluorescens *and *P. putida *is predicted from genome context analysis (see text, Fig. 8); the formed reaction product is not known (-).

**Figure 8:**
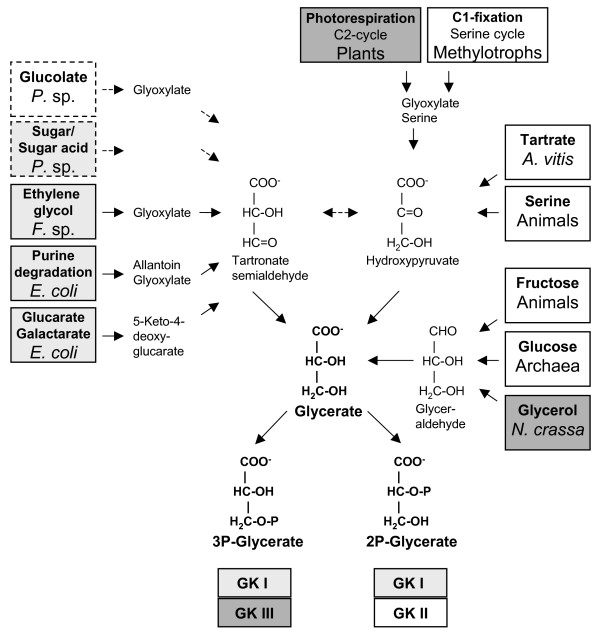
**The key role of glycerate kinase in carbohydrate metabolism**. Current knowledge about the physiological function of glycerate kinases of the different glycerate kinase classes in extant living organisms is shown (according to Table 3). The different carbon sources/pathways and organisms are boxed and the color indicates the involvement of glycerate kinases of the three different glycerate kinase classes (class II GK (MOFRL family), white; class I GK, light grey; class III GK, dark grey) in the respective metabolic pathway. The formation of 2- or 3-phosphoglycerate is indicated by the name of the enzyme family indicated below the compounds. The suggested function of the class I and class II (MOFRL family) glycerate kinases in *Pseudomonas fluorescens *and *P. putida *is indicated by dashed lines.

In order to confirm the presence of the branched ED pathway in *T. tenax *and beyond that in other hyperthermophilic Archaea and in order to study this pathway's regulation, the gene encoding the putative glycerate kinase was cloned and the enzymatic and regulatory properties of the gene product were characterized. Here we describe the first hyperthermophilic archaeal glycerate kinase, the enzyme of *Thermoproteus tenax*. Moreover, new data from comparative genomics analyses as well as available biochemical information indicate a much broader distribution of the branched ED pathway in Archaea than previously assumed. Finally, the evolution of glycerate kinases was re-evaluated revealing the presence of three independent glycerate kinase classes that share no common origin: i) the glycerate kinase class I (GK I), ii) the glycerate kinase class II (GK II, MOFRL family) as well as iii) a novel glycerate kinase family related to the phosphoribulokinase/uridine kinase family, here named glycerate kinase class III (GK III). The affiliation of enzymes with the different enzyme classes, their phylogenetic distribution as well as their physiological function are discussed. This study confirms the complexity and mosaic nature of the central carbohydrate metabolic pathways in extant organisms.

## Results and discussion

Combined genomics and biochemical studies suggested the presence of the branched ED pathway in *Thermoproteus tenax *[10]. In the genome of *T. tenax*, one gene homolog with high similarity to predicted glycerate kinases (COG 2379, EC 2.7.1.31) and hydroxypyruvate reductases (glycerate dehydrogenases) (COG 2379, EC 1.1.1.29 (NADH), EC1.1.1.81 (NAD(P)H)) was identified [8]. In order to confirm the predicted glycerate kinase activity and thus the presence of the branched ED pathway in *T. tenax *and to gain first insights into its regulation as well as its physiological role, the encoding gene was expressed as a recombinant protein in *E. coli*, purified and characterized.

### Cloning of the garK gene, expression and purification of the glycerate kinase from *T. tenax*

**Figure 2:**
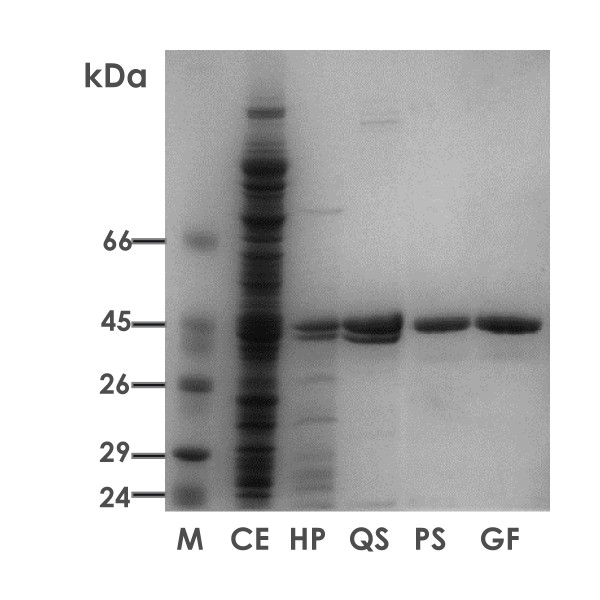
**Purification of the recombinant class II glycerate kinase of *T. tenax***. SDS-PAGE of the recombinant expression and purification of *T. tenax *glycerate kinase. Lanes with crude cell extracts (CE, 20 μg), soluble fractions after heat precipitation (HP, 10 μg) and after purification by Q sepharose (QS), phenyl sepharose (PS) and gel filtration (GF) (5 μg) are shown. 'M' refers to the protein marker, Dalton Mark VII-L (Sigma)).

The gene encoding the putative glycerate kinase/hydroxypyruvate reductase from *T. tenax *(*garK*, AJ621354) revealed an open reading frame of 1197 bp coding for a polypeptide of 398 amino acids. The glycerate kinase/hydroxypyruvate reductase homolog revealed significant overall similarity with the recently characterized glycerate kinase of the thermoacidophile *P. torridus*, (27% identity, Blastp search) as well as numerous "annotated" glycerate kinases and hydroxypyruvate reductases or glycerate dehydrogenases in all three domains of life. The *garK *gene was cloned and expressed in *E. coli *BL21 (DE3) using the pET expression vector system. The recombinant enzyme was enriched from *E. coli *crude extract by heat precipitation at 80°C for 30 minutes. Further purification was achieved by Q-sepharose, phenyl sepharose and gel filtration (Fig. 2). From 10 g wet cells of recombinant *E. coli*, 2.72 mg of homogenous glycerate kinase with a specific activity of 4.6 U/mg protein was recovered.

The glycerate kinase of *T. tenax *migrated as a single band on SDS-PAGE with an apparent molecular mass of 44 kDa (Fig. 2), corresponding well to the calculated mass of 42.4 kDa. The molecular mass of the native enzyme was determined by gel filtration on a HiLoad 26/60 Superdex 200 prep grade column and revealed 48.3 ± 1.1 kDa, indicating a monomeric structure for the glycerate kinase of *T. tenax*. A monomeric structure was also reported for the characterized *Hyphomicrobioum methylovorum *GM2 glycerate kinase [33] and the enzyme of *T. acidophilum *[30]. Interestingly, biochemical data suggest a dimeric structure for the glycerate kinase of *P. torridus *as well as *Thermotoga maritima *[32,34], although analysis of the crystallographic packing of the *T. maritima *enzyme (TM1585) indicates a monomeric structure [34]. Also many plant enzymes (e.g. *Brassica campestris *[35], spinach leaf [36]), which, however, as discussed later are members of a different glycerate kinase class, exhibit a monomeric structure.

### Enzymatic and regulatory properties of the glycerate kinase from T. tenax

Glycerate kinase activity was determined in a discontinuous assay at 70°C by coupling the formation of 2-phosphoglycerate with NADH oxidation in the presence of enolase, pyruvate kinase and L-lactate dehydrogenase. Glycerate kinase catalyzed the ATP-dependent phosphorylation of D-glycerate yielding 2-phosphoglycerate. No formation of 3-phosphoglycerate was observed in response to the addition of phoshoglycerate mutase to the assay. The enzyme showed activity only in the presence of ATP and Mg^2+^. No activity was detected in controls without protein or without the (co-)substrates D-glycerate, ATP or both D-glycerate and ATP. The enzyme showed no hydroxypyruvate reductase activity either in the presence of NADH + H^+ ^or NADPH + H^+^.

**Figure 3:**
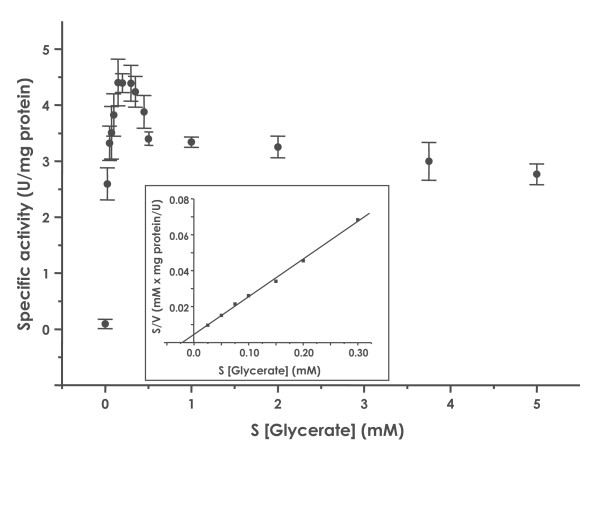
**Kinetic properties of the class II glycerate kinase of *T. tenax***. The glycerate kinase activity was determined in a discontinuous assay at 70°C by coupling the formation of 2-phosphoglycerate with NADH oxidation via enolase, pyruvate kinase and lactate dehydrogenase. The dependence of the specific enzyme activity on the glycerate concentration is shown. The enzyme is inhibited at higher glycerate concentrations. The insert shows the linear transformation according to Hanes for glycerate concentrations up to 0.3 mM.

The 2-glycerate kinase activity was measured in the presence of different substrate and co-substrate concentrations. Strikingly, the enzyme was inhibited at higher glycerate concentrations (Fig. 3) and apparent K_m_- and V_max_-values for glycerate (0.02 ± 0.01 mM, 5.05 ± 0.52 U/mg protein, D-glycerate at concentrations below 0.3 mM) and for ATP (K_m _of 0.03 ± 0.01 mM, V_max _of 4.41 ± 0.04 U/mg protein) at 70°C were estimated. No substrate inhibition was observed for the enzymes of *P. torridus*, *T. acidophilum*, and *H. methylovorum*. As shown in Table 1, the *T. tenax *enzyme exhibits a higher substrate affinity, however, the specific activity is significantly reduced in comparison to the glycerate kinases of *P. torridus *and *H. methylovorum*. This is reflected in an about 6 or 7-fold (glycerate) and 5 or 9-fold (ATP) reduced catalytic efficiency (k_cat_/K_m_-values, Tab. 1) of the hyperthermophilic enzyme compared to *P. torridus *and *H. methylovorum*, respectively. The glycerate kinase of *T. acidophilum *shows a similar catalytic efficiency for glycerate, only for ATP it is slightly (2.3-fold) increased.

**Table 1:** Comparison of kinetic and biochemical properties from characterized class II glycerate kinases (MOFRL family)

		**Archaea**			**Bacteria**
		
		**Crenarchaea**	**Euryarchaea**		
		
		***T. tenax***^1^	***P. torridus***^2^	***T. acidophilum***^3^	***H. methylovorum***^4^
**Growth optimum (°C)**		90	60	59	28
**Molecular mass**					
Subunit (kDa)		44	50	45	52
Native (kDa)		48,3	95	49,3	41
Calculated (kDa)		42,4	46,6	45,8	(46.5)^5^
Oligomeric structure		Monomer	Dimer	Monomer	Monomer
**Kinetic Parameters**					
D-Glycerate	K_m _(mM)	(0.02)^6^	0,34	(0.56)^7^	0,13
	V_max _(U mg^-1^)	(5.05)^6^	435	(118)^7^	181
	k_cat _(min^-1^)	214	20271	5404	8417
	k_cat_/K_m _(min^-1^mM^-1^)	8913	59621	9651	64742
ATP	K_m _(mM)	(0.03)^6^	0,51	(0.23)^7^	0,13
	V_max _(U mg^-1^)	(4.41)^6^	432	(118)^7^	181
	k_cat _(min^-1^)	187	20131	5404	8417
	k_cat_/K_m _(min^-1^mM^-1^)	7187	39473	16889	64742
**Reaction product**		2-PG	2-PG	2-PG	2-PG
**Temperature optimum (°C)**		90	60	70	50
**Substrate specificity (%)**					
L-Glycerate		30	34	nd	13
**Phosphate donor specificity (%)**					
ATP		100	100	100	100
GTP		20	37	0–4	59
CTP		16	24	0–4	59
UTP		10	39	0–4	64
TTP		16	nd	nd	nd
**Metal ion specificity (%)**					
Mg^2+^		100	100	100	100
Co^2+^		56	11	8	75
Mn^2+^		59	11	10	72
Ni^2+^		30	25	0	29
Ca^2+^		15	nd	0	0
**Inhibition by ADP**		+	nd	nd	+

^1^this study; ^2 ^[32]; ^3 ^[30] ^4 ^[33]; ^5^since no information of the encoding gene is available, the mean value of subunit and native molecular mass was formed and used for the calculation of the k_cat_/K_m _value; ^6^since glycerate inhibition is observed for the *T. tenax *enzyme the estimated values for glycerate concentration up to 0.3 mM are given; ^7^determined for DL-Glycerate; nd, not determined; +, detected.

The ability of the *T. tenax *glycerate kinase to phosphorylate other compounds than D-glycerate was tested in a discontinous assay at 70°C by monitoring the formation of ADP from the ATP-dependent phosphorylation of substrate via pyruvate kinase and lactate dehydrogenase. The following compounds were substituted for D-glycerate: the stereoisomer L-glycerate, sugar acids (galactonate, gluconate, malate, pyruvate, lactate), glyceraldehyde, glycerol, serine and phosphorylated intermediates (3-phosphoglycerate, glyceraldehyde 3-phosphate (GAP)). The enzyme is absolutely specific for glycerate as phosphoryl acceptor. Like the enzymes of *P. torridus *[32], *H. methylovorum *[33] and plant [37] glycerate kinases described so far, the enzyme showed higher activity with D-glycerate (100%) and only low activity with L-glycerate (30%) (Tab. 1).

The *T. tenax *glycerate kinase has a temperature optimum at 90°C (100% activity), correlating well with its growth optimum (around 90°C). At 70°C only 55% and at 50°C only 15% residual activity were observed.

The specificity of the enzyme for phosphoryl donors (5 mM) was tested for ATP, CTP, GTP, TTP, UTP, ADP as well as PP_i _and polyphosphates (P_3_, P_5 _and P_25_). The enzyme exhibited highest activity with ATP, although ATP could be partially replaced by CTP, GTP, TTP and UTP (16%, 20%, 16% and 10% activity, respectively). No activity was observed with ADP, PP_i _and polyphosphates.

The glycerate kinase of *T. tenax *required divalent metal ions for activity and the highest activity was observed in the presence of 20 mM Mg^2+^. Activity was inhibited by the addition of EDTA (40 mM). Co^2+^, Mn^2+ ^and Ni^2+ ^(all 5 mM) and Ca^2+ ^(2 mM) could partially replace Mg^2+ ^(56%, 59%, 30% and 15% activity, respectively). No activity was detected in the presence of Cu^2+ ^and Fe^2+ ^at 0.2, 2, 5 and 20 mM. Thus, in respect of cosubstrate specificity and dependence on metal ions, the enzyme of *T. tenax *resembles the enzymes of *P. torridus *and *H. methylovorum *and plant glycerate kinases described previously [32,33,38] (Tab. 1). Monovalent ions (K^+ ^and NH_4_^+^), which were reported to activate the *H. methylovorum *glycerate kinase [33], showed no effect on the *T. tenax *and *P. torridus *enzyme.

Effector studies were performed in the presence of non-saturating concentrations of D-glycerate (50 μM) and non-saturating (50 μM) and saturating (5 mM) concentrations of ATP. The reaction rate of *T. tenax *glycerate kinase showed no significant alteration by the following intermediates (1 and 10 mM): AMP, intermediates of the EMP pathway (glucose, glucose 6-phosphate, fructose 1,6-bisphosphate, fructose 6-phosphate, dihydroxyacetone phosphate, GAP, 3-phosphoglycerate, lactate), the branched ED pathway (galactonate, gluconate, KDG), glycogen metabolism (glucose 1-phosphate) and the citric acid cycle (citrate, isocitrate, α-ketoglutarate, succinate, fumarate, malate). The reaction product ADP showed an inhibitory effect on the glycerate kinase activity. In the presence of non-saturating concentrations of ATP, the inhibition by ADP was much higher suggesting competitve inhibition (non-saturating ATP: 76%, 39.1%, 8.2% and saturating ATP: 93.2%, 87.3%, 42% activity of control at concentrations of 0.01, 0.1, and 1 mM ADP, respectively; the average over two independent measurements is given). A similar inhibition by ADP is also reported for the *H. methylovorum *enzyme [33]. For the *P. torridus *and *T. acidophilum *enzyme respective studies were not performed [32].

In summary, the gene product of the *garK *gene possesses exclusively 2-glycerate kinase activity, and no 3-glycerate kinase or hydroxypyruvate reductase activity was detected. The first characterized archaeal hyperthermophilic enzyme exhibits common features with the well characterized enzymes of *P. torridus, T. acidophilum *and *H. methylovorum *such as (co)substrate specificity and metal ion dependence. At the same time, it also shows some unique characteristics such as its inhibition by glycerate and its hyperthermophilic properties (Tab. 1). The physiological significance of the inhibition by glycerate is unknown, since information about the cellular concentrations of glycerate in *T. tenax *is currently unavailable. The key enzyme of the non-phosphorylative branch of the ED pathway in *T. tenax *seems to be no subject of broad regulatory control, at least for the effectors tested, but seems to be regulated by the energy charge of the cell probably via competitve (product) inhibition by ADP.

### Distribution of the branched ED pathway in Archaea

New comparative genomics based data [12,13] and available biochemical information revealed a much broader distribution of the branched pathway in Archaea than previously assumed (Tab. 2). As supported by biochemical and phylogenetic data, the branched ED pathway is not only present in hyperthermophilic Archaea (e.g. *T. tenax*, Sulfolobales) but represents the pathway for sugar degradation in thermoacidophilic Archaea (e.g. Thermoplasmatales) and Haloarchaea, with the only exception of *Halobacterium sp. *NRC-1.

**Table 2:** Phylogenetic distribution of ED key enzymes in Archaea utilizing the ED pathway

Enzyme		**GAD**	**KD(P)GA**	**GK II**	**KDGK**	
Enzyme family		MR-MLE	NAL	MORFL	PfkB	BadF/BadG/BcrA/BcrD
EC		4.2.1.39	4.1.2.-	2.7.1.31	2.7.1.45	2.7.1.59
COG		4948	0329	2379	0524	2971
**Crenarchaea**						
Thermoproteales	TTX	1156 AJ621281	1156a AJ621282	0788 AJ621345	1157 AJ621283	
Sulfolobales	SACI	0885	0225	0113	0226	
	SSO	3198	3197	0666	3195	
	STO	2366	2479	2037	2478	
**Euryarchaea**						
Halobacteriales	HMA	3069	0207	7015	0545	
	HQ	2412A	1507A	1667A	1455A	
	VNG	0442G	0444G	-	0158G	
	NPH	0998A	1490A	1162A	3184A	
Thermoplasmatales	PTO	0485	1026	1442	-	0011
	TA	0085	0619	0453m	-	0122
	TVN	179275	663048	797109	-	204668
	FAC	0084	1067	0418	-	1438

Blast searches were performed with the characterized *T. tenax *enzymes or the *T. acidophilum *KDG kinase. The gene numbering is according to [67]. The table represents an update of recently published work referring to the phylogenetic distribution of genes involved in glucose and pentose metabolism in Archaea [13].

Characterized enzymes are underlined. Abbreviations: GAD, gluconate dehydratase; KD(P)GA, 2-keto-3-deoxy-(6-phospho)-gluconate aldolase; GK II, glycerate kinases class II; KDGK, 2-keto-3-deoxygluconate kinase. EC, Enzyme Commission; COG, Clusters of Orthologous Groups. MR-MLE, mandelate racemase/muconate lactonizing enzyme subgroup of the enolase superfamily; NAL, N-acetylneuraminate lyase superfamily; MORFL, multi-organism fragment with rich leucine family; PfkB, ribokinase-like superfamily, pfkB family carbohydrate kinase; BadF/BadG/BcrA/BcrD, BadF/BadG/BcrA/BcrD ATPase family

FAC: *Ferroplasma acidarmanus fer1*, HMA: *Haloarcula marismortui ATCC 43049*, HQ: *Haloquadratum walsbyi *DSM16790, NPH: *Natronomonas pharaonis DSM 2160*, PTO: *Picrophilus torridus DSM 9790*, SACI: *Sulfolobus acidocaldarius DSM 639*, SSO: *Sulfolobus solfataricus P2*, STO: *Sulfolobus tokodaii str. 7*, TA: *Thermoplasma acidophilum DSM 1728*, TTX: *Thermoproteus tenax*, TVN: *Thermoplasma volcanium GSS1*, VNG: *Halobacterium sp. NRC-1*

In thermoacidophiles the specific enzymes of the npED branch glyceraldehyde dehydrogenase (*P. torridus *[19], *T. acidophilum *[19,39]) as well as the glycerate kinase (*P. torridus *[32], *T. acidophilum *[30]) have been characterized recently. In addition, the presence of the spED branch was demonstrated by the identification of a novel KDG kinase in *T. acidophilum *[39], which is not related to the characterized enzymes of *T. tenax *and *S. solfataricus *(ribokinase-like superfamily, pfkB family carbohydrate kinase, PF00294), but is a member of the BadF/BadG/BcrA/BcrD ATPase family (PF01869). Homologs of the new KDG kinase were identified in all members of the Thermoplasmatales with available genome sequence information. In Haloarchaea the presence of glycerate kinase homologs in *Haloarcula marismortui*, *Haloquadratum walsbyi *and *Natronomonas pharaonis *suggests the presence of the branched ED pathway rather than the assumed spED pathway. Therefore the branched ED pathway – rather than the semi- or non-phosphorylative ED pathway – seems to be common for sugar degradation in Archaea that utilize the ED pathway.

### Phylogenetic analyses

#### Glycerat kinase class II (MOFRL family)

**Figure 4:**
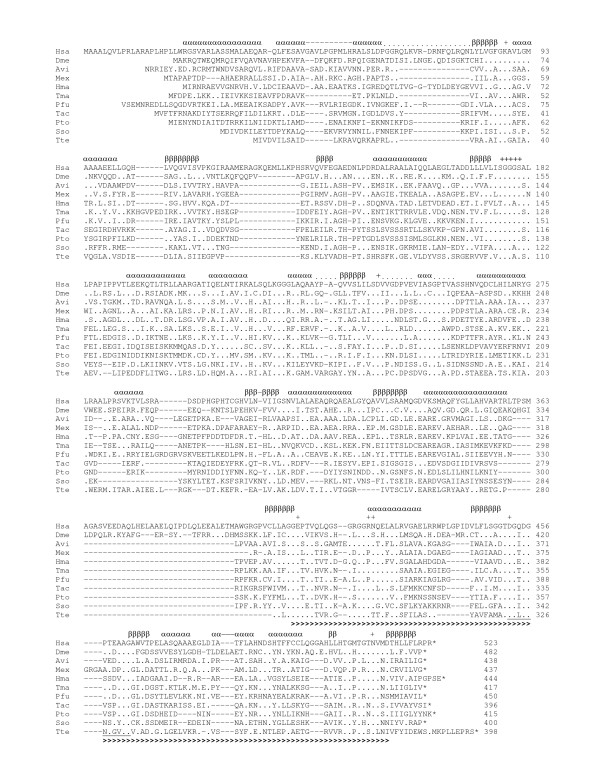
**Multiple sequence alignment of class II glycerate kinases (MOFRL family)**. The determined secondary structure of the *Thermotoga maritima *enzyme is shown above the sequences and the catalytic sites are marked (+). Amino acids identical to the first sequence are replaced by a dot; this allows the simple visualization of conserved and divergent regions. The determined consensus motif (PRATT, (D-X(0,2)-G-X(0,1)-D-[GP]-X(4)-[APS]-[ACDGST] (residues 322–332 of the *T. tenax *glycerate kinase)) is underlined and the MOFRL domain (*T. maritima *residue 304–410) is indicated by a bold ">"sign. Abbreviations: Avi, *Agrobacterium vitis*; Dme, *Drosophila melanogaster*; Hma, *Haloarcula marismortui*; Hsa, *Homo sapiens*; Mex, *Methylobacterium extorquens*; Pfu, *Pyrococcus furiosus*; Pto, *Picrophilus torridus*; Sso, *Sulfolobus solfataricus*; Tac, *Thermoplasma acidophilum*; Tma, *Thermotoga maritima*; Tte, *Thermoproteus tenax*.

**Figure 6:**
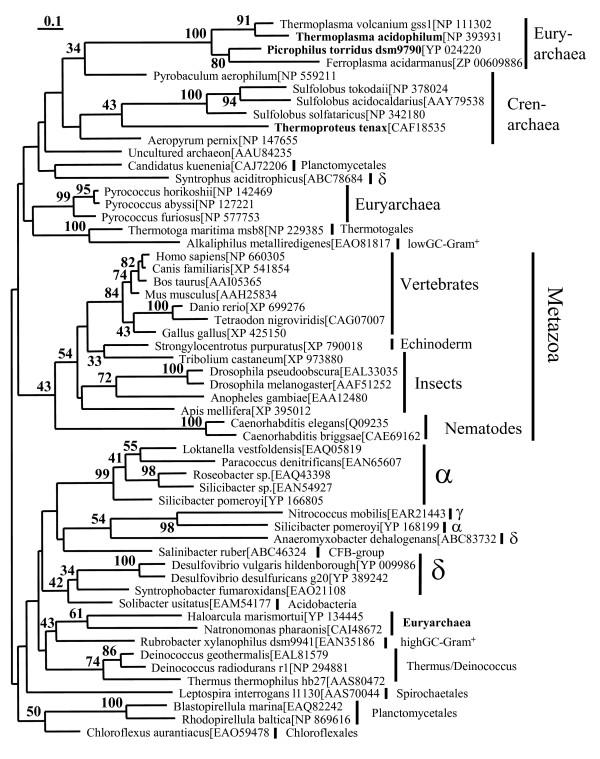
**Phylogenetic analyses of class II glycerate kinases (MOFRL family)**. The best Maximum Likelihood tree based on 56 sequences and 169 positions inferred by the program Treefinder with a WAG+Γ_4 _model. Numbers at internal nodes are corresponding to the bootstrap support values obtained in 100 replicates (using the same program and model). Only values above 30% are indicated. Characterized enzymes are indicated in bold.

Data bank searches (BlastX, BlastP) revealed sequences homologous to the glycerate kinase of *T. tenax *in all three domains of life: Bacteria, Eukarya and Archaea. In Archaea, glycerate kinase homologs were identified in about half (19 of 35) of the sequenced genomes (Tab. 2, Fig. 4, Fig. 6). Beside Archaea that utilize the ED pathway for sugar degradation (Tab. 2) homologs were also identified in the genomes of *Aeropyrum pernix *(APE0996), *Pyrobaculum aerophilum *(PAE1309), *Thermofilum pendens *(TPE0207), *Metallosphaera sedula *(MSED0161) and in Thermococcales (*Pyrococcus horikoshii *(PHO0495), *P. abyssi *(PAB1021), *P. furiosus *(PFU0024), *Thermococcus kodakarensis *(TKO1893)). No glycerate kinase homologs were identified in the genomes of *Halobacterium *sp. NRC-1, *Pyrobaculum islandicum*, *Archaeoglobus fulgidus*, *Nanoarchaeum equitans *and Methanogens (*Methanocaldococcus jannaschii*, *Methanococcoides burtonii*, *Methanococcus maripaludis*, *Methanoculleus marisnigri*, *Methanopyrus kandleri*, *Methanosaeta thermophila*, *Methanosarcina acetivorans*, *M. barkeri, M. mazei*, *Methanosphaera stadtmanae*, *Methanospirillum hungatei*, *Methanothermobacter thermautotrophicus*).

Whereas all archaeal and eukaryal organisms harbor only one gene encoding glycerate kinase, paralogous genes that originated in gene duplications and/or horizontal gene transfer (HGT) were found in several bacteria (e.g. *Ralstonia solanacearum*, *Sinorhizobium meliloti *(Fig. 7)). All homologs are characterized by the conserved C-terminal MORFL (multi-organism fragment with rich leucine) domain (residues 304–410 of the glycerate kinase of *T. maritima*, TM1585). For the identification of conserved amino acid patterns sequences of all identified members of class II glycerate kinases (MOFRL family) were analyzed by PRATT 2.1 [40,41] revealing one conserved motif (D-X(0,2)-G-X(0,1)-D-[GP]-X(4)-[APS]-[ACDGST] (residues 322–332 of the *T. tenax *glycerate kinase: DGLDGNTGVAG).

**Figure 5:**
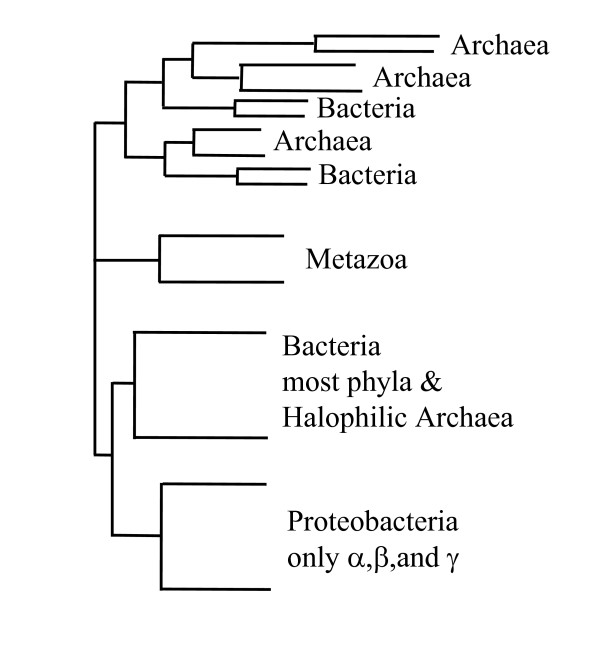
**Phylogenetic analyses of class II glycerate kinases (MOFRL family)**. Cartoon illustrating the global tree topology. Best Maximum Likelihood trees are shown in Fig. 6 and 7.

In order to analyze the phylogenetic relationship between the GK II members, we aligned 135 sequences. An overview of their relationships is shown in form of a cartoon (Fig. 5). From this group 56 sequences were selected, which adequately represent the original diversity. After the elimination of highly divergent regions, a total of 169 amino acid residues were used for the construction of the phylogenetic tree shown in Fig. 6. Phylogenetic analyses based on Maximum Likelihood resulted in a complex tree topology with three major clades. Bootstrap analyses show a low support for all basal branches.

**Figure 7:**
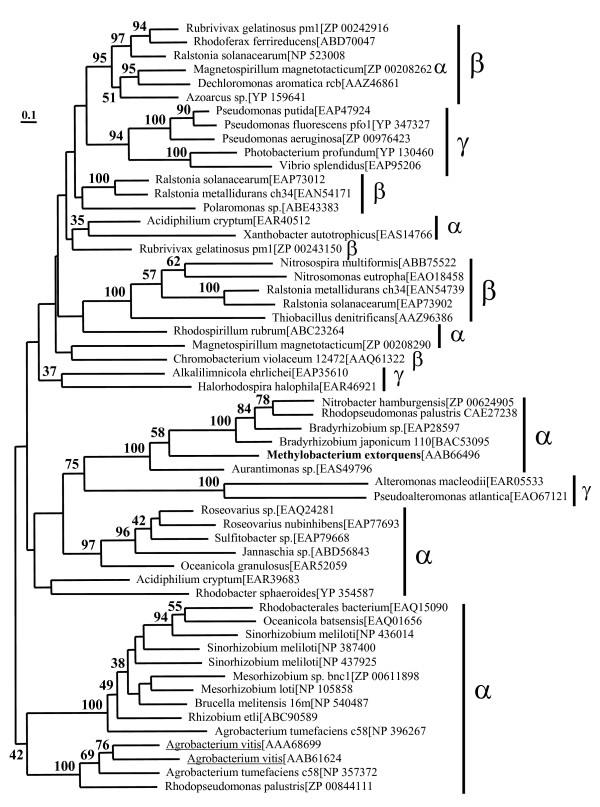
**Phylogenetic analyses of class II glycerate kinases (MOFRL family)**. The best Maximum Likelihood tree based on 56 more closely related proteobacterial sequences (α-, β- and γ-Proteobacteria) and 374 amino acid positions inferred by Treefinder. The MOFRL homologs of *Agrobacterium vitis*, which were predicted to exhibit hydroxypyruvate reductase activity are underlined. All other manipulations are identical to the one described in Fig. 6.

Clade I consists essentially of archaeal homologs with a few basal branching bacteria. Within this group several lineages display a pronounced acceleration, namely the Thermoplasmatales and the Sulfolobales together with *Thermoproteus*. More precisely, the archaeal clade I is divided in two lineages: one with euryarchaeal (Thermoplasmatales), crenarchaeal as well as two bacterial homologs (*Candidatus kuenenia*, *Syntrophus aciditrophicus*) and a second lineage with euryarchaeal (*Pyrococcus *sp.) and again two bacterial homologs(*T. maritima*, *Alkaliphilus metalliredigenes*).

The second clade consists exclusively of animals. It represents the only basal branch that is weakly supported. The clade II is divided in a major group of vertebrates, an echinoderm and several insects, which are separated from the very fast-evolving nematodes. Since it is highly likely that these bilaterian animals form a monophyletic group, this observation reveals the limited resolving power of the data set and supports the view that the other groups are substantially older than the animals.

The third clade consists almost exclusively of bacterial sequences and represents the major group of bacterial homologs. However, it includes two haloarchaeal sequences possibly due to a HGT event with a high GC-Gram positive. This group represents a fair amount of the bacterial diversity (ten deeply branching lineages/phyla). Nevertheless, the great majority of the sequenced bacterial genomes do not contain homologous sequences.

The presence of bacterial and archaeal sequences in the first clade can be interpreted in two different ways. Either there was an early gene duplication event that created two copies in Archaea and Bacteria and subsequently these copies were lost in most lineages, or this gene was originally present in Archaea and at least two HGT events from Archaea to Bacteria created the current distribution. The phylogenetic tree contains representatives of all three domains of life. Consequently, it seems likely that the glycerate kinase is truly universal. This notion is supported by the fact that, despite the presence of possible HGT events at a later time, the three domains are essentially discrete. One of the lineage-specific HGT events is found in the case of the high GC-Gram positive *Rubrobacter xylanophilus*. This bacterium likely represents the donor lineage (Clade III) for the glycerate kinase enzymes present in the two extreme halophilic Archaea *Haloarcula marismortui *and *Natronomonas pharaonis *(Euryarchaea). No homolog was identified in the closely related *Halobacterium *NRC-1 genome. Further evidence for HGT events between Archaea and Bacteria in the context of thermoadaptation was demonstrated for the hyperthermophile *T. maritima *[42].

Within the bacterial clade, there is a group that is limited to more closely related proteobacterial sequences (α-, β-, γ-group). The Maximum Likelihood phylogenetic tree of this group is shown in Fig 7. It is based on 56 sequences and 374 amino acid residues. Since these sequences are much closer related, there are about two times the number of positions that can be used in the phylogenetic analyses. The proteobacterial sequences are divided in two basic lineages. One of them consists exclusively of α-Proteobacteria and a mixed group of α-, β- and γ-Proteobacteria. In Bacteria – in contrast to Eukarya and Archaea – several species were identified with two or multiple glycerate kinase isoenzymes, representing examples of early (e.g. *Ralstonia solanacearum*) or more recent gene duplications (e.g. *Sinorhizobium meliloti*). The mosaic picture in extant Proteobacteria might therefore represent the result of a complex mixture of gene duplications with subsequent differential losses and/or potential lateral gene transfer elements.

#### Three distinct glycerate kinase classes

In contrast to previous reports [31,32], PSI Blast analyses revealed that members of the glycerate kinase class II (MOFRL family) show no similarity to members either of the bacterial glycerate kinase class I (GK I) or the recently identified novel kinase family (here named glycerate kinase class III, GK III) [31]. Therefore, known glycerate kinases divide in three distinct, unrelated glycerate kinase classes and seem to represent examples of independent (non-homologous) functional evolution. Interestingly, PSI Blast analyses of class III glycerate kinases revealed a diverse superfamily with enzymes that catalyze substantially different enzyme reactions such as glycerate kinase, phosphoribulokinase (EC 2.7.1.19), panthotenate kinase (EC 2.7.1.33), uridine kinase (EC 2.7.1.48) and are involved in cell division (cell division recognition particle). These data suggest that class III glycerate kinases are members of the diverse phosphoribulokinase/uridine kinase family (PF00485, nucleoside/nucleotide kinase (NK) superfamily cd02019) within the P-loop kinases, which phosphorylate all kind of different substrates [43].

The crystal structures of three glycerate kinases, one GK class I (*Neisseria meningitidis *(PDB|1to6)) and two GKs class II (MOFRL family, *Pyrococcus horikoshii *(PH0495; PDB|1x3l, *T. martima *(TM1585, PDB|2b8n), [34]) are established. As reported previously by Schwarzenbacher et al. [34] structural similarity search revealed no significant structural similarity between the two glycerate kinase structures of *N. meningitides *(class I) and *T. maritima *(class II). So far no glycerate kinase class III structure is available. However, several structures of different P-loop kinases have been established, which are structurally unrelated to class I or II glycerate kinases. Therefore, the glycerate kinases of *A. thaliana *and *S. cerevisiae *represent a third class of glycerate kinases. In summary available protein structures reveal three different structural classes of glycerate kinases, which are structurally unrelated and thus evolutionarily distinct.

### Physiological function and affiliation of characterized enzymes with the three different glycerate kinase classes

The identification of three distinct glycerate kinase classes raises questions about the affiliation of previously characterized enzymes with the different enzyme classes and more generally about the distribution and physiological function in extant organisms (Tab. 3, Fig. 8). Respective analyses remain difficult since many biochemical studies go back to the 1950–1970s and often the genes encoding these enzymes were not identified.

Members of the **class II glycerate kinases **(MOFRL family, PF05161; GckA/TtuD-like family (PDB: 1o0u, SCOP)) were identified in all three domains of life (19 Archaea, 75 Bacteria, 16 Eukarya; IPR 007835). They represent the only glycerate kinases identified in Archaea and animals (Metazoa; vertebrates, echinoderm, insects, nematodes). In Bacteria a preferred presence in α-, γ- and δ-Proteobacteria is observed. The glycerate kinase of *T. tenax*, the first characterized archaeal hyperthermophilic enzyme (this study), and of the thermoacidophiles *P. torridus *[32] and *T. acidophilum *[30] represent the only archaeal enzymes characterized so far. Several bacterial and eukaryal members have been characterized previously, although their affiliation with a new enzyme classes was not considered.

In Bacteria, class II glycerate kinase has been identified in several facultative methylotrophes, where the enzyme is a constituent of the serine cycle for conversion of C1-compounds (e.g. methane, methanol) in C3-compounds. Glycerate kinases of *Methylobacterium extorquens *[44] and *H. methylovorum *GM2 [33] were examined and shown to form 2-phosphoglycerate. The serine pathway and significant levels of glycerate-2-kinase were also reported in *Pseudomonas *species grown on methanol as well as oxalate [45-47].

In different strains of *Agrobacterium vitis *(AB3, AB4), a grapevine pathogen, two plasmid-encoded GK II homologs (*ttuD*4 (pTrAB4), *ttuD*3 (pTrAB3)) were identified. The genes are part of the plasmid-encoded tartrate utilization gene clusters. Using a mutational approach, hydroxypyruvate reductase activity was suggested although the catalyzed reaction was unknown [48,49]. The tartrate utilization pathway shares common reactions with the serine cycle (conversion of hydroxypyruvate via 2-phosphoglycerate to pyruvate). From the absence of similarities of TtuD to known hydroxypyruvate reductases and the high similarity to glycerate kinase it was already previously suggested that the enzyme encodes glycerate kinase rather than hydroxypyruvate reductase [44]. Therefore, in contrast to annotations found in many genome sequencing projects and data banks (COG, SCOP and Pfam), so far only glycerate kinases and no hydroxypyruvate reductases or glycerate dehydrogenases were identified as members of class II glycerate kinases (MOFRL family).

In animals, class II glycerate kinase is the key enzyme for gluconeogenesis from serine and is involved in fructose metabolism [50,51]. Glycerate kinases have been characterized in great detail from different organisms and organs (e.g. rat liver, kidney cortex [52,53]) and all enzymes characterized so far were shown to be specific for 2-phosphoglycerate formation. Therefore, all class II glycerate kinases (MOFRL family) characterized until now seem to be specific for 2-phophoglycerate formation.

Members of the classical **class I glycerate kinase (GK I) **(Pfam 02595; glycerate kinase I (PDB: 1to6, SCOP)) represent the major glycerate kinase in Bacteria and few Eukarya (220 Bacteria, 7 Eukarya (*Entamoeba histolytica *(2), *Trypanosoma cruzi *(2), *Fusarium graminearum*, *Aspergillus nidulans*, *Dictyostelium discoideum*; IPR004381). No homolog was identified in Archaea and animals and only few homologs in α-, and δ-Proteobacteria. However, many homologs were found in β- and γ-Proteobacteria as well as low- and high-GC Gram positives. This is nicely complementary since there are almost no Gram positives that harbor class II glycerate kinases (MOFRL enzyme family), whereas the α-, and δ-Proteobacteria do contain predominantly members of class II glycerate kinases.

Glycerate kinases have been characterized from different bacterial sources. In *E. coli *(K12) two different glycerate kinases were identified, which are subject to independent biosynthetic regulation [54]. In more recent studies it was shown that the two glycerate kinases are members of the GK I family (class). The two encoding genes were identified in conserved gene clusters and their involvement in allantoin metabolism (purine degradation via glyoxylate and the glycerate pathway; GK-1, *glxK *gene, [55]) and glucarate and galactarate utilization (GK-2, *gclK *gene, [56]) was demonstrated. Both pathways share the conversion of tartronate semialdehyde (TSA) catalyzed by TSA reductase to glycerate and the phosphorylation via glycerate kinase (Fig. 8). Whereas the enzyme involved in sugar acid degradation forms 2-phosphoglycerate, the enzyme of the glycerate pathway forms 3-phosphoglycerate. Phylogenetic analyses indicate that also the *Flavobacterium *sp. glycerate kinase (*Leeuwenhoekiella blandensis *MED217, EAQ51383), which was purified from ethylene glycol grown cells and uses the glycerate pathway for conversion to pyruvate via 2-phosphoglycerate [57], is a member of this enzyme family. Therefore, the currently characterized enzymes of the class I glycerate kinases form either 2- or 3-phosphoglycerate.

Members of a **novel third glycerate kinase class (named GK class III here) **were reported recently (IPR 006083, PF00485, COG 4240 predicted kinases; P-loop-containing NTP hydrolases (PDB: 1a7j, SCOP)) [31]. Detailed phylogenetic analysis revealed a more general distribution in cyanobacteria (e.g. most *Synechococcus *sp., *Prochlorococcus marinus*) as well as in proteobacteria, especially of the γ-group (e.g. *Nitrosococcus oceanii*, *Pseudoalteromonas tunicate*) than reported previously (data not shown). Therefore, this enzyme family seems to occur in fungi, plant and few bacteria (i.e. most cyanobacteria and few γ-proteobacteria). Some of the sequences in the databanks were annotated as phosphoribulokinase/uridine kinase due to the presence of the PRK/UK domain (IPR 006083, Pfam 00485, amino acid 210–425 *A. thaliana*) and their affiliation with the diverse phosphoribulokinase/uridine kinase family (see above). Interestingly, in cyanobacteria two *Synechococcus *strains (JA-2-3B'a (ABD02569), JA-3-3Ab (ABC99586)) as well as *Synechocystis *sp. Pcc6803 (P73408) possess only a class I glycerate kinase homolog.

The enzymes of *Arabidopsis thaliana *(*At1g80380*) and *Saccharomyces cerevisiae *(NP_011721) were shown to possess glycerate kinase activity and were characterized [31]. Both enzymes are specific for glycerate and form 3-phosphoglycerate. Many plant enzymes were characterized in great detail previously, but the encoding genes were not identified [31,35-38]. In plants, glycerate kinase plays an important role in the photorespiratory C-2 cycle, which compensates for the oxygenase activity of ribulose-1,5-bisphosphate carboxylase/oxygenase (RUBISCO) and thus serves as carbon recovery system reconverting 2-phosphoglycolate to 3-phosphoglycerate [31]. The C-2 cycle involves at least ten reaction steps localized in different cell organelles (chloroplast, peroxisome, mitochondria). The final step of glycerate phosphorylation is performed in the chloroplast.

In fungi, a role of glycerate kinase was reported in *Neurospora crassa *grown on glycerol [58], which is metabolized by a phosphorylative pathway via glycerol kinase and an oxidative pathway via NADP^+^-glycerol dehydrogenase, glyceraldehyde dehydrogenase and glycerate kinase. Phylogenetic analysis revealed only one homolog of the class III glycerate kinases in *Neurospora crassa *(EAA32802). The function in Bacteria (Cyanobacteria and Proteobacteria) remains unclear and a role in carbon metabolism, as reported for fungi [58], or in photorespiration, as suggested for complex cyanobacteria [31], might be possible.

In summary, phylogenetic analyses of class II (MOFRL), class I and class III glycerate kinases revealed a separated distribution: (i) class II members in Archaea, animals and Bacteria, especially α-, β- and γ-Proteobacteria, (ii) class I members in Bacteria (especially Gram positives and β- and γ-Proteobacteria, but only few Cyanobacteria) and (iii) members of class III glycerate kinases in plants, fungi, Cyanobacteria and few Proteobacteria (many of the γ-group). Interestingly, no organism harbors members of all three glycerate kinase classes and only very few comprise members of two different glycerate kinase classes. *Pseudomonas fluorescens *and *P. putida *harbor a class II glycerate kinase (MOFRL) homolog as well as the class I glycerate kinase. In addition, few fungi (*Coccidioides immintis*, *Aspergillus nidulans*, *Giberella zeae*) comprise class I and class III glycerate kinase homologs. The physiological role of the different glycerate kinase homologs is still unclear. For the facultatively methylotrophic *Pseudomonas *species a putative role of the GK class II homolog in C1-fixation as well as glyoxylate metabolism and for the GK class I homolog in sugar/sugar acid degradation might be suggested [45-47] (Tab. 3, Fig. 8). This is supported by the organization of the GK class II gene homolog (PFL1595) in a gene cluster comprising genes encoding pyruvate kinase (PFL1594), tartronate semialdehyde reductase (TSAR) (PFL1596), hydroxypyruvate isomerase (PFL1597) and glyoxylate carboligase (tartronate-semialdehyde synthase) (PFL1598) in *Pseudomonas fluorescens *(strain Pfo-1 and Pf-5) as well as *P. putida*. A similar co-organisation of the class II glycerate kinase (MOFRL family) and pyruvate kinase is also reported in *M. extorquens *and *A. vitis *and a function in the generation of pyruvate/acetyl-CoA for anabolic purposes is discussed [44]. The GK class I gene homolog (PFL2908) is found in a gene cluster with a putative transcriptional regulator involved in sugar acid recognition (PFL2909), a putative 2-hydroxyacid dehydrogenase (PFL2904) and a putative PfkB family carbohydrate kinase (putative KDG kinase; PFL2902).

As shown in Fig. 8, there seems to be no correlation between the biochemical function or pathway and the enzyme involved from the metabolic point of view. For example, sugar acids (e.g. glucarate) are degraded in *E. coli *via a class I glycerate kinase and in *T. tenax *glucose degradation via gluconate is performed by a member of class II glycerate kinases. However, these analyses demonstrate that glycerate is a key metabolite in the central carbohydrate metabolism. Glycerate is formed during the degradation of various compounds such as amino acids (serine), sugars (glucose, fructose), sugar acids (glucarate, oxalate, tartrate), glycerol, as well as during the synthesis of C-3 compounds from C-1 (methane, methanol) and C-2 (2-phosphoglycolate) compounds. The different metabolic routes merge at the level of the two isomers hydroxypyruvate and tartronate-semialdehyde as well as glyceraldehyde, which are converted to glycerate via redox reactions. Therefore, the conversion of glycerate to 2- or 3-phosphoglycerate by glycerate kinases of the three different enzyme classes is the key reaction for channeling a great variety of intermediates into the EMP pathway, which serves as funnel for catabolic as well as anabolic purposes. So far, not much is known about the metabolism of carbohydrates other than glucose, galactose and fructose in Archaea. These findings suggest that Archaea share with organisms from other domains of life the common concept that alternative metabolic routes channel into the EMP pathway via the glycerate kinase reaction.

## Conclusion

To our knowledge, this report contains the first characterization of a hyperthermophilic archaeal glycerate kinase. The re-evaluation of available archaeal genome sequence information revealed that the branched Entner-Doudoroff (ED) pathway, rather than the suggested non- or semi-phosphorylative ED pathway, is common for sugar degradation in Archaea that utilize the ED pathway (with the only exception of *Halobacterium *spec. NRC-1). Archaeal glycerate kinases are members of the MOFRL (multi-organism fragment with rich leucine) family and in contrast to many current annotations found, so far no hydroxypyruvate reductases or glycerate dehydrogenases were identified as members of this enzyme family. Detailed phylogenetic studies demonstrated the presence of three distinct glycerate kinase classes that share no common origin, and are distributed separately in extant organisms. The affiliation of characterized glycerate kinases with the three different enzyme classes as well as their physiological function gives no evidence to the association with particular pathways. However, our results highlight the key function of glycerate kinase in funneling various substrates into the common EMP pathway for catabolic and anabolic purposes.

## Methods

### Strains and growth conditions

Cultures of *T. tenax *(DSM 2078, [14] were grown as reported previously [17]. *E. coli *strains DH5α (Life Technologies), BL21(DE3) (Novagen) for cloning and expression studies were grown under standard conditions [59] following the instructions of the manufacturer.

### (Bio)chemicals and enzymes

If not indicated otherwise, (bio)chemicals and enzymes were purchased from Sigma-Aldrich, VWR International or Roche Diagnostics GmbH in analytical grade. D- and L-Glycerate was purchased from Sigma.

### Heterologous expression

For heterologous expression, the pET vector system (pET-24a, Novagen) was used. The *garK *gene coding for the putative glycerate kinase, (TTX_0788, AJ 621345) was cloned into pET-24a via two new restriction sites (N*de*I and E*co*RI) intoduced by PCR mutagenesis with the primer set Ttx-garK-pETf 5'GTTGCAAGTCGACTACCATATGATAG3' and Ttx-garK-pETrev 5'TATACTTGGAATTCCTCCTCC3'. PCR mutagenesis was performed using *Pfu *polymerase (Fermentas) and genomic DNA from *T. tenax *as template. The sequence of the cloned gene was confirmed by dideoxy sequencing of both strands. Expression of the recombinant enzyme in *E. coli *BL21(DE3) was performed following the instructions of the manufacturer (Novagen).

### Protein purification

Recombinant *E. coli *cells (10 g wet weight) were suspended in 20 ml of 100 mM HEPES/KOH (pH 7.0) containing 7.5 mM dithiothreitol (buffer A) and passed three times through a French pressure cell at 150 MPa. Cell debris and unbroken cells were removed by centrifugation (60 000 × *g *for 30 min at 4°C). For enrichment, the resulting crude extract was diluted 1:1 with buffer A and subjected to a heat precipitatation at 80°C for 30 min. After heat precipitation, the sample was cleared by centrifugation (60 000 × *g *for 30 min at 4°C), dialyzed overnight against 50 mM HEPES/KOH (pH 7.0), 7.5 mM dithiothreitol (2-liter volume, 4°C). Enzyme purification was achieved by chromatography on Q sepharose fast flow (Amersham Biosciences) pre-equilibrated in 50 mM HEPES/KOH (pH 7.0), 7.5 mM dithiothreitol. All glycerate kinase activity was found in the run-through fraction. (NH_4_)_2_SO_4 _was added to the protein fraction to a final concentration of 0.7 M and the mixture was applied to a phenyl sepharose (high performance) column (Amersham Biosciences) pre-equilibrated in buffer A containing 0.7 M (NH_4_)_2_SO_4_. After three washing steps with buffer A, protein was eluted by a linear gradient of buffer A with increasing concentration of ethylene glycol (0–100%). Fractions with glycerate kinase activity were pooled and dialyzed overnight against 50 mM HEPES/KOH (pH 7.0), 7.5 mM dithiothreitol, 300 mM KCl. After concentration by membrane filtration (Vivaspin 6 ml Concentrator, 5000-MWCO PES, Vivascience Sartorius Group) protein was subjected to gel filtration on HiLoad 26/60 Superdex 200 prep grade (Amersham Biosciences) pre-equilibrated in dialysis buffer. Fractions containing the homogeneous enzyme fraction were pooled and used for enzymatic assays. Protein concentration was determined according to Bradford [60] using the reagent kit from BioRad Laboratories (Munich, Germany) and bovine serum albumin as standard. The purity and molecular mass of the subunits of glycerate kinase were examined by SDS-polyacrylamide gel electrophoresis under denaturating conditions according to Laemmli [61] with Dalton Mark VII-L (Sigma) as standard.

### Molecular mass determination

The native molecular mass was determined by gel filtration on a HiLoad 26/60 Superdex 200 prep grade column (Amersham Biosciences) using the same running conditions as for enzyme purification (protein concentration: 0.45 mg protein/ml). Standards: ferritin type I (horse spleen, 443 kDa), alcohol dehydrogenase (yeast, 148 kDa), D-lactate dehydrogenase (*Lactobacillus leichmanii*, 78 kDa), and cytochrome c (bovine heart, 12.5 kDa).

### Enzyme assays and determination of kinetic, enzymatic and regulatory properties

#### Glycerate kinase assay

For the determination of all parameters, except substrate specificity, a discontinuous assay at 70°C, which monitors the glycerate-dependent formation of 2-phosphoglycerate was used. In the standard assay the phosphorylation of glycerate by ATP was followed by coupling the formation of the reaction product 2-phosphoglycerate to the oxidation of NADH via enolase (*S. cerevisiae*, bakers yeast EC 4.2.1.11), pyruvate kinase (rabbit muscle, EC 2.7.1.40) and lactate dehydrogenase (rabbit muscle, EC 1.1.1.27). The formation of 3-phosphoglycerate was studied by the addition of 1 unit of phosphoglycerate mutase (rabbit muscle, EC 5.4.2.1). The glycerate kinase assay was performed in 100 mM HEPES/KOH (pH 7.5, 70°C) in the presence of glycerate kinase (10 μg protein/ml volume), 5 mM ATP and 20 mM MgCl_2_. The reaction was started by the addition of 0.25 mM D-glycerate. The indicator reaction (0.5 ml total volume) was performed at 37°C in 100 mM HEPES/KOH (pH 7.5, RT), 10 mM MgCl_2_, 5 mM ADP, 0.5 mM NADH, 3.5 units of pyruvate kinase, 2.5 units of lactate dehydrogenase and 100 μl aliquots from the glycerate kinase assay. The reaction was started by addition of 1 unit enolase. Enzymatic activities were measured by monitoring the increase in absorption at 340 nm (ε_NADH _= 6.3 mM^-1 ^cm^-1^). The measured enzyme activity was directly proportional to the amount of enzyme added to the assay. If not stated otherwise, three independent measurements were performed for each assay, and the experimental error is given.

For determination of K_m_- and V_max_-values for D-glycerate and ATP, concentration ranges of 0–5 mM and 0–10 mM, respectively, in the presence of 20 mM Mg^2+ ^were used. In order to assure the linearity of the enzyme reaction kinetics, reaction times of 0, 1, 2 and 3 min were recorded for each measurement. Calculation of the kinetic parameters (V_max _and K_m_) were performed by iterative curve-fitting (Hill) using the program Origin (Microcal Software Inc.).

Co-substrate specificity was tested by substituting ATP (5 mM) for alternative phosphoryl donors (CTP, GTP, TTP, UTP, ADP as well as PP_i _and P_3_, P_5 _and P_25 _polyphoshates) at equimolar concentrations. Measurements were generally performed in the presence of 20 mM Mg^2+^; for PP_i _and polyphosphate 1 mM Mg^2+ ^was added. Metal ion requirement was analyzed by exchanging Mg^2+ ^for other divalent metal ions and by addition of EDTA. Samples were incubated at 70°C for 0 and 3 minutes and ion concentrations of 0.2, 2, 5 and 20 mM were tested. EDTA was added at a final concentration of 40 mM in the presence of 20 mM Mg^2+^. The effects of K^+ ^and NH_4_^+ ^were studied at concentrations of 50 mM.

For effector studies, activity was determined in the presence of non-saturating concentrations of D-glycerate (0.05 mM) as well as non-saturating (0.05 mM) or saturating (5 mM) concentrations of ATP. Temperature dependence was followed over a wide range of temperatures (30–100°C) using the standard glycerate kinase assay.

Substrate specificity was measured at 70°C in a discontinuous assay by monitoring ADP formation from the ATP-dependent phosphorylation of different substrates. ADP formation was coupled to the oxidation of NADH via pyruvate kinase and lactate dehydrogenase. The glycerate kinase assay was performed as described above but in the presence of alternative substrates (5 mM) instead of the D-glycerate. The indicator reaction (0.5 ml total volume) was performed at 37°C in 100 mM HEPES/KOH (pH 7.5, RT), 10 mM MgCl_2_, 5 mM phosphoenol-pyruvate, 0.5 mM NADH, 3.5 units of pyruvate kinase and 2.5 units of lactate dehydrogenase. The reaction was started by addition of 100 μl aliquots from the glycerate kinase assay. Enzymatic activities were determined as described above.

#### Hydroxypuruvate reductase/glycerate dehydrogenase assay

Hydroxypyruvate reductase activity was determined at 70°C using a continuous assay. The reduction of hydroxypyruvate to glycerate was followed in the presence of NAD(P)H + H^+ ^in 100 mM HEPES/KOH (pH 7.5, RT) including 10 mM hydroxypyruvate, 0.5 mM NAD(P)H + H^+ ^and 10 μg of protein (assay volume 1 ml).

### Sequence Handling and phylogenetic analyses

The initial alignments, obtained with CLUSTAL X [62], were manually refined using the ED option of the MUST program package [63]. All data sets were analysed by a Maximum Likelihood (ML) method, implemented in Treefinder [64], with a model based on the Whelan and Goldman (WAG) matrix of amino acid replacements assuming a proportion of invariant positions and gamma distributed rates (WAG+F+I+Γ4). All phylogenetic trees presented are Treefinder ML topologies. Bootstrap analyses with 100 replicates were used to estimate the support for internal nodes of the phylogenies (Treefinder, WAG+F+I+Γ4 model). The bootstrap consensus tree was subsequently generated by the CONSENSE option of the PHYLIP package [65]. PSI Blast analyses were performed using the NCBI browser [66].

## Authors' contributions

DK carried out the cloning, expression, purification and biochemical characterization of the glycerate kinase of *T. tenax*. HA contributed to experimental design, enzyme purification and characterization, data mining (comparative genomics studies) and to the writing process. HB performed all phylogenetic studies. BS conceived of the study, designed and coordinated the study, carried out data mining and prepared the manuscript draft. All authors read and approved the final manuscript.
